# A Novel *ARMC5* Germline Variant in Siblings With Primary Bilateral Macronodular Adrenal Hyperplasia and Colonic Adenomas

**DOI:** 10.1210/jcemcr/luaf197

**Published:** 2025-08-29

**Authors:** Takahisa Handa, Hiraku Kameda, Yuki Oe, Kiyoshi Sakai, Akinobu Nakamura, Tatsuya Atsumi

**Affiliations:** Department of Internal Medicine, Kushiro Red Cross Hospital, Kushiro 085-8512, Japan; Department of Endocrinology and Diabetes, JCHO Hokkaido Hospital, Sapporo 062-8618, Japan; Department of Rheumatology, Endocrinology and Nephrology, Faculty of Medicine, Graduate School of Medicine, Hokkaido University, Sapporo 060-8638, Japan; Department of Internal Medicine, Kushiro Red Cross Hospital, Kushiro 085-8512, Japan; Department of Rheumatology, Endocrinology and Nephrology, Faculty of Medicine, Graduate School of Medicine, Hokkaido University, Sapporo 060-8638, Japan; Division of Diabetes and Endocrinology, Department of Medicine, NTT Sapporo Medical Center, Sapporo 060-0061, Japan; Department of Internal Medicine, Kushiro Red Cross Hospital, Kushiro 085-8512, Japan; Department of Rheumatology, Endocrinology and Nephrology, Faculty of Medicine, Graduate School of Medicine, Hokkaido University, Sapporo 060-8638, Japan; Department of Rheumatology, Endocrinology and Nephrology, Faculty of Medicine, Graduate School of Medicine, Hokkaido University, Sapporo 060-8638, Japan

**Keywords:** primary bilateral macronodular adrenal hyperplasia, armadillo repeat containing 5 (*ARMC5*), colonic adenoma, mild autonomous cortisol secretion

## Abstract

We report 2 siblings with primary bilateral macronodular adrenal hyperplasia (PBMAH). Both case 1, a 61-year-old male, and case 2, his 54-year-old brother, presented with incidentally discovered multiple nodules in bilateral adrenal glands on computed tomography (CT) scan. There was no family history of endocrine disease nor visible signs of Cushing syndrome. Early morning adrenocorticotropic hormone (ACTH) levels were low, and a dexamethasone suppression test revealed unsuppressed cortisol, leading to a diagnosis of PBMAH with mild autonomous cortisol secretion (MACS). Examinations for extra-adrenal lesions detected colorectal adenomas in both patients. Genetic analysis revealed a novel germline pathogenic variant (c.2647del) in the armadillo repeat containing 5 (*ARMC5*) gene in both cases. Because both patients preferred not to undergo surgery, we opted to follow their condition with periodic imaging studies, including CT and colonoscopy. Our experience suggests that *ARMC5* gene pathogenic variants are associated with colorectal tumors as well as PBMAH. Therefore, periodic screening with colonoscopy should be considered in patients with PBMAH.

## Introduction

Primary bilateral macronodular adrenal hyperplasia (PBMAH) is present in less than 2% of cases with Cushing syndrome and is characterized by multiple large hyperplastic nodules in the bilateral adrenal glands and varying levels of excess cortisol [1]. Most cases are diagnosed between 45 and 65 years of age. Many cases are diagnosed incidentally through radiographic imaging studies. In PBMAH, cases presenting with mild autonomous cortisol secretion (MACS) are more frequent than cases presenting with overt Cushing syndrome [2].

Variants of the armadillo repeat containing 5 (*ARMC5*) gene have been reported with high frequency in PBMAH [3, 4]. The *ARMC5* gene is involved in the induction of apoptosis and regulation of the cell cycle. In patients with PBMAH, pathogenic variants in the *ARMC5* gene are associated with the development of meningiomas [5]. In addition, complications, such as colorectal cancer, breast cancer, thyroid cancer, and parathyroid tumors, have been reported in patients with PBMAH [4]. Germline variants in the *ARMC5* gene are found in approximately 80% of the PBMAH patients with familial history [6], highlighting the importance of considering familial risk during diagnostic evaluation. In sporadic cases of PBMAH, variants in the *ARMC5* gene are reported to be found in approximately 20% to 25% of cases [7, 8]. Here, we report 2 siblings with PBMAH and colonic adenomas harboring a novel germline pathogenic variant (c.2647del) of the *ARMC5* gene.

## Case Presentation

### Case 1

**Figure 1.**
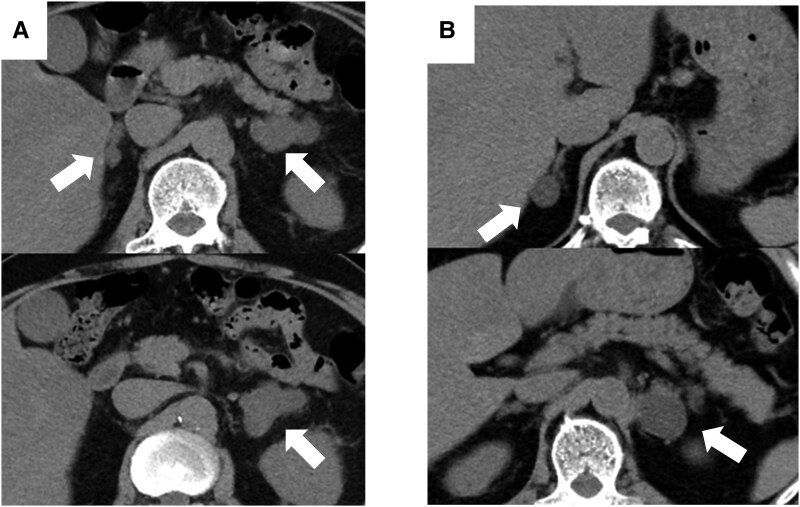
Adrenal nodules. (A) Abdominal computed tomography (CT) scan of case 1. (B) Abdominal CT scan of case 2. Arrows indicate adrenal nodules.

A 61-year-old man who underwent a computed tomography (CT) scan during a COVID-19 infection was found to have multiple nodules on the bilateral adrenal glands and was referred to our department (Fig. 1A). He had a history of hypertension treated with amlodipine and candesartan, and benign prostatic hypertrophy treated with silodosin and tadalafil. He did not report any known familial history of endocrine disorders, but his maternal uncle had gastric cancer. He was 157.9 cm tall, weighed 62.8 kg, and had no visible signs of Cushing syndrome. His ambulatory blood pressure was 126/82 mmHg.

### Case 2

A 51-year-old man, the younger brother of case 1, was referred to our department after bilateral adrenal nodules were found on a CT scan performed for evaluation of abnormal liver function (Fig. 1B). He had a history of sleep apnea syndrome, steatotic liver disease, intestinal hernia, and hypertension treated with olmesartan. His systolic blood pressure at home was high at approximately 150 mmHg. He was 160.7 cm tall, weighed 61.7 kg and had no visible signs of Cushing syndrome.

## Diagnostic Assessment

### Case 1

Early morning adrenocorticotropic hormone (ACTH) levels were low (<1.5 pg/mL [<0.33 pmol/L]; normal reference range: 7.2-63.3 pg/mL [1.58-13.93 pmol/L]), and a dexamethasone suppression test revealed unsuppressed cortisol (5.6 µg/dL [154.56 nmol/L]; normal reference range: <1.8 µg/dL [<49.68 nmol/L]). Serum cortisol measured at 11:00 Pm was high (5.2 µg/dL [143.52 nmol/L]; normal reference range: <5.0 µg/dL [<138.0 nmol/L]). 24- hour urinary cortisol excretion was normal. There were no abnormalities in lipid metabolism. He was diabetic with a fasting blood glucose level of 130 mg/dL(7.22 mmol/L; normal reference range: 70-110 mg/dL [3.89-6.11 mmol/L]; Table 1). Based on the characteristic imaging phenotype and hormonal evaluation, the patient was diagnosed with PBMAH and MACS. He was simultaneously diagnosed with diabetes based on elevated fasting blood glucose levels.

**Table 1.** Laboratory findings

	Case 1	Case 2	Reference range
Fasting plasma glucose	**130 mg/dL** **(7.22 mmol/L)**	96 mg/dL (5.33 mmol/L)	70-110 mg/dL (3.89-6.11 mmol/L)
HbA1c (NGSP)	6.2%	6.1%	4.6-6.2%
PRA	0.6 ng/mL/ hours (0.6 µg/L/ hours)	<0.2 ng/mL/ hours (<0.2 µg/L/ hours)	0.2-2.3 ng/mL/ hours (0.2-2.3 µg/L/ hours)
PAC	2.52 ng/dL (0.07 nmol/L)	1.2 ng/dL (0.03 nmol/L)	0.4-8.21 ng/dL (0.01-0.23 nmol/L)
Serum DHEA-S	28 µg/dL (0.76 µmol/L)	**14 µg/dL** **(0.38 µmol/L)**	Case 1: 24-244 µg/dL (0.65-6.59 µmol/L) Case 2: 38-313 µg/dL (1.03-8.45 µmol/L)
<early morning> Serum ACTH	**<1.5 pg/mL** **(<0.33 pmol/L)**	**3.5 pg/mL** **(0.77 pmol/L)**	7.2-63.3 pg/mL (1.58-13.93 pmol/L)
<early morning> Serum cortisol	9.8 µg/dL (270.48 nmol/L)	7.0 µg/dL (193.2 nmol/L)	7.07-19.6 µg/dL (195.13-540.96 nmol/L)
<23:00> Serum ACTH	**<1.5 pg/mL** **(<0.33 pmol/L)**	N/A	
<23:00> Serum cortisol	**5.2 µg/dL** **(143.52 nmol/L)**	N/A	
<1 mg overnight DST> Serum cortisol	**5.6 µg/dL** **(154.56 nmol/L)**	**7.3 µg/dL** **(201.48 nmol/L)**	
24 hours urine cortisol	29.7 µg/24 hours (81.97 nmol/day)	N/A	4.3-176 µg/24 hours (11.87-485.76 nmol/day)
Spot urinary metanephrine creatinine ratio	72.3 µg/gCre (366.56 nmol/gCre)	57.6 µg/gCre (292.03 nmol/gCre)	40-190 µg/gCre (202.8-963.3 nmol/gCre)
Spot urinary normetanephrine creatinine ratio	217 µg/gCre (1184.39 nmol/gCre)	130 µg/gCre (709.54 nmol/gCre)	90-330 µg/gCre (491.22-1801.14 nmol/gCre)

Values in parenthesis are International System of Units (SI). Abnormal values are indicated in bold. Items not tested are indicated as N/A.

Abbreviations: ACTH, adrenocorticotropic hormone; DHEA-S, dehydroepiandrosterone sulfate; DST, dexamethasone suppression test; HbA1c, glycated hemoglobin; PAC, plasma aldosterone concentration; PRA, plasma renin activity.

**Figure 2.**
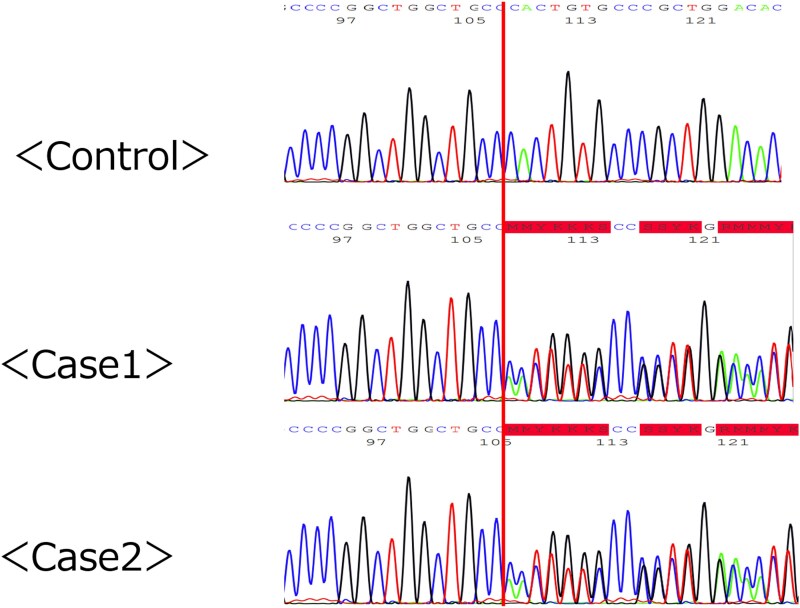
Genetic analysis in blood samples of siblings. The red line indicates a single nucleotide deletion (c.2647del) in exon 6 found in the armadillo repeat containing 5 (*ARMC5*) gene. This pathogenic variant results in a frameshift change in all amino acids after 823 of the ARMC5 protein.

Bone density evaluation revealed a T-score (lumbar spine: 0.6, femur: −0.7) within the normal range. During examination for extra-adrenal lesions, colonoscopy identified 2 adenomas in the ascending colon. Genetic analysis revealed a single nucleotide deletion (c.2647del) in exon 6 of the *ARMC5* gene, a previously unreported germline pathogenic variant (Fig. 2). We also excised and examined a colon polyp, which showed no somatic variant, only a germline pathogenic variant.

### Case 2

Early morning ACTH levels were low (3.5 pg/mL [0.77 pmol/L]; normal reference range: 7.2-63.3 pg/mL [1.58-13.93 pmol/L]), and a dexamethasone suppression test revealed unsuppressed cortisol (7.3 µg/dL [201.48 nmol/L]; normal reference range: <1.8 µg/dL [<49.68 nmol/L]), leading to the diagnosis of PBMAH with MACS. He had no evidence of lipid metabolic abnormalities. Hemoglobin A1c (HbA1c) was 6.1% (normal reference range: 4.6%–6.2%), suggesting potential mild abnormality of glucose metabolism (Table 1). Bone density was normal (T-score: lumbar spine: 0.2, femur: −1.0). Examination for extra-adrenal lesions by colonoscopy revealed multiple adenomas in the ascending and transverse colon. Genetic analysis showed the same germline pathogenic in the *ARMC5* gene as in case 1 (Fig. 2). Similar to his sibling, only a germline pathogenic variant was found in the resected colorectal polyp from the patient in case 2.

## Treatment

In both cases, given the patients' preference to forgo surgery and their mild metabolic conditions, we opted to monitor the patients by periodic imaging studies.

## Outcome and Follow-Up

Both cases have shown no increase in adrenal tumor size, worsening of metabolic disorders, or new extra-adrenal lesions for 18 months.

## Discussion

We present 2 siblings with PBMAH and a novel germline pathogenic variant in the *ARMC5* gene. Notably, both patients developed colorectal adenomas, suggesting a potential link between *ARMC5* germline pathogenic variants and colorectal adenoma development. *ARCM5* pathogenic variants are believed to cause PBMAH according to the two-hit hypothesis, that is, that adrenal tumorigenesis is triggered by a somatic variant in the other allele, in addition to a germline pathogenic variant [3]. The ARMC5 protein is composed of 935 amino acids and contains 2 distinct regions: armadillo repeats and a broad-complex, tramtrack, and bric-à-brac (BTB) domain [9]. The BTB domain is located between amino acids 748 and 816 of ARMC5 and is close to the variant site identified in these cases. ARMC5 binds to ubiquitin ligase cullin-3 (CUL3) via the BTB region and degrades sterol regulatory element-binding protein (SREBP) to maintain cholesterol homeostasis. Variants in ARMC5 increase SREBP, which leads to increased cholesterol synthesis and cell proliferation, resulting in tumorigenesis [10]. Cases of PBMAH with variants near the BTB domain, as in the present cases, have been reported [7]. These variants are thought to impair SREBP degradation, leading to adrenal tumor formation.

Meningiomas, which can be associated with PBMAH, are similarly suggested to develop through a second hit mechanism in the *ARMC5* gene. Previous reports have documented the co-occurrence of extra-adrenal tumors, such as breast cancer, parathyroid tumors, thyroid cancer, and thyroid adenomas, in individuals with *ARMC5* variants [4]. Unlike meningiomas, the role of *ARMC5* variants in these lesions, which do not exhibit second hits or loss of heterozygosity, remains unclear. Since the possibility of an association between *ARMC5* pathogenic variants and extra-adrenal lesions cannot be excluded, we believe patients with PBMAH should be screened for other tumorous lesions. Therefore, we performed imaging studies, including colonoscopy, of these patients. A link between *ARMC5* variants and the development of colorectal adenomas has not been reported. The genetic analysis of colorectal adenomas in both siblings revealed a germline pathogenic variant in the *ARMC5* gene, without evidence of additional somatic variants. A Japanese study reported a colorectal polyp prevalence of 16.5%. The probability of a sibling also having colorectal polyps is relatively low (approximately 2.7%) [11]. Therefore, while a chance occurrence is possible, the involvement of an *ARMC5* gene pathogenic variant cannot be excluded. More studies are needed to determine the hypothesized association between PBMAH and colonic adenomas.

In PBMAH with excessive cortisol secretion, various complications, including diabetes, hypertension, dyslipidemia, osteoporosis, and steatotic liver disease, have been reported [12]. While the frequency remains unknown, the coexistence of sleep apnea syndrome with PBMAH has also been reported [13]. Here, we should note that both patients presented with metabolic abnormalities (hypertension and diabetes in case 1, and hypertension, steatotic liver disease, and sleep apnea in case 2). Management of metabolic abnormalities is based on the guidelines for each disease.

As both patients declined surgical intervention and the excess cortisol and associated metabolic abnormalities were mild, a conservative approach to follow-up was chosen in both cases. In patients with bilateral adrenal incidentalomas presenting with subclinical Cushing syndrome, unilateral adrenalectomy has been reported to improve hypertension, osteoporosis, diabetes, or impaired glucose tolerance [14]. As bilateral adrenalectomy requires lifelong glucocorticoid replacement therapy and carries the risk of adrenal crisis and over-replacement, unilateral adrenalectomy of the larger adrenal tumor is typically considered for PBMAH with MACS. Therefore, in this case, unilateral adrenalectomy should be considered if metabolic abnormalities associated with hypercortisolemia worsen in the future.

## Learning Points

- A novel *ARMC5* germline pathogenic variant (c.2647del) may be associated with the development of PBMAH.
- Colorectal adenomas might be associated with *ARMC5* gene pathogenic variants in PBMAH.
- To screen for extra-adrenal lesions, colonoscopy should be considered during PBMAH follow-up.

## Data Availability

Some or all datasets generated and/or analyzed during the current study are not publicly available but are available from the corresponding author upon reasonable request.

## Abbreviations

ACTH: adrenocorticotropic hormone
BTB: broad-complex, tramtrack, and bric-à-brac
CT: computed tomography
MACS: mild autonomous cortisol secretion
PBMAH: primary bilateral macronodular adrenal hyperplasia
SREBP: sterol regulatory element-binding protein
